# Determinants of academic performance among medical students in Egypt: a multi-center cross-sectional study

**DOI:** 10.3389/fpubh.2025.1710562

**Published:** 2026-01-07

**Authors:** Mohamed Saad Sayed, Yusof Mohamed Omar, Naira A. Shoman, Mohab Sherif, Ursula Abu Nahla, SES Collaborative Group, Doaa Mahmoud Khalil

**Affiliations:** 1 Faculty of Medicine, Beni-Suef University, Beni Suef, Egypt; 2 Faculty of Medicine, Mansoura University, Mansoura, Egypt; 3 Faculty of Medicine, Helwan University, Helwan, Cairo, Egypt; 4 Faculty of Medicine, Hebron University, Hebron, Palestine; 5 Public Health and Community Medicine, Faculty of Medicine, Beni-Suef University, Beni Suef, Egypt

**Keywords:** academic performance, medical students, Egypt, socioeconomic status, institutional factors

## Abstract

**Background:**

Academic performance is a critical determinant of career opportunities for medical students. This study aimed to identify the socio-demographic, institutional, and social factors associated with academic achievement among medical students across Egypt's diverse university systems.

**Methods:**

A multi-center cross-sectional study utilizing an online survey was conducted from January to May 2025, involving 751 medical students from 38 public, private, and Al-Azhar universities across Egypt. Academic performance was measured using self-reported cumulative percentage. Multivariable linear regression was used to analyze the relationships between academic performance and various predictive factors.

**Results:**

The mean cumulative percentage for the cohort was 85 (SD = 8.96). In the final multivariable model, lower academic performance was significantly associated with being male (unstandardized coefficient (*B*) = −1.77, 95% CI = −3.03, −0.52), attending an Al-Azhar university compared to a public one (B = −5.75, 95% CI = −8.23, −3.26), and living in student housing vs. with family (*B* = −3.31, 95% CI = −4.68, −1.94). Conversely, higher academic performance was significantly associated with receiving parental support (*B* = 2.06, 95% CI = 0.03, 4.08) and being from a medical school in the Lower Egypt region compared to Urban Governorates (*B* = 1.94, 95% CI = 0.49, 3.38).

**Conclusion:**

Academic performance among Egyptian medical students is significantly influenced by a combination of institutional, demographic, and social support factors, which appear more impactful than traditional socioeconomic markers in this cohort. These findings highlight potential systemic inequities and emphasize the need for targeted educational interventions and policy review to ensure a fair and supportive learning environment for all students.

## 1 Introduction

Academic performance is the cornerstone of success in higher education and can have long-lasting implications on students' future career prospects and professional competence. This is particularly true for medical education, where academic performance during medical school often determines the future specialty choices available to students and the quality of training programs they are likely to be accepted into (1, 2).

However, the determinants of academic performance are often multifaceted, and various factors play a role in shaping the academic success of medical students throughout medical school (3). One such influencing factor is socioeconomic status, which includes various factors such as income, parental education, and occupation (4). Students who enjoy the financial support of their families, or have family members within the healthcare profession who can guide them early on, often have a much easier time adapting to the pressures of medical education and are thus more likely to perform better academically (5).

However, the relationship is not always straightforward. Systematic reviews reveal a complex web of influencing factors that extend beyond socioeconomic status to include psychological well-being, institutional environments, and individual student lifestyle choices (3, 6). These claims are further evident in the variability of results within studies conducted in similar regional contexts. For instance, a recent study on Sudanese medical students found that high family income was significantly associated with higher academic achievement (7), a finding supported by another study in Ethiopia (8), which linked lower monthly allowance to poor performance. In contrast, a study was also conducted in Sudan (9), found no significant association between family income and GPA. Similarly, maternal education was identified as a critical determinant of performance (8), as well as the father's education (9). On the other hand, other studies found no significant association with either parent's education level, further complicating the picture (7).

In recent years, medical students in Egypt have had to face mounting economic pressures, which contributed to widespread unhealthy behaviors and psychological challenges (10). Their environment was further reshaped when the medical education system was fundamentally transformed in 2018, shifting from a traditional, six-year teacher-centered program to an integrated, five-year competency-based curriculum with earlier clinical exposure (11). Such a drastic transition can exacerbate existing maladaptive lifestyles and psychological stressors, potentially undermining academic performance. Furthermore, the reform was not implemented equally across universities in Egypt due to various constraints, leading to a lack of uniformity in both curricula and examinations across medical schools (11). This lack of uniformity is particularly consequential for the equity of postgraduate residency selection in Egypt, as the current system directly compares student scores from different institutions without accounting for the systemic variability in curricula and assessment rigor.

While some national studies have explored predictors of related constructs like academic self-efficacy in light of these recent changes (12), there is a limited number of studies investigating the changing educational landscape and the direct institutional and socioeconomic determinants of academic performance across diverse university systems in Egypt. Given the importance of academic success in shaping the career opportunities of the future medical workforce, it is essential to understand the complex interplay of factors that influence student performance. This study aims to fill the gap by assessing the predictors of academic achievement in Egypt. This study intends to provide insights for educational institutions to develop targeted support systems that address specific student needs, foster a more equitable learning environment, and ultimately enhance the quality of medical training accessible to future healthcare providers in Egypt.

## 2 Methods

### 2.1 Study design

This multi-center cross-sectional study was conducted between January and May 2025, following the STROBE guidelines (13).

### 2.2 Study population and sample size

The study population comprised undergraduate medical students from 38 universities across Egypt, including 23 public, 12 private/national, and 3 Al-Azhar universities, selected proportionately to the national distribution of medical schools (14). Participants were proportionally recruited from all major regions of Egypt: Urban Governorates (41.7%), which include major metropolitan areas such as Cairo and Alexandria; Lower Egypt (38.1%), encompassing the Nile Delta region north of Cairo; Upper Egypt (18.2%), which includes the Nile Valley region south of Cairo; and the Frontier Governorates (2.0%), the large governorates bordering neighboring countries (14). Eligible participants were those enrolled in their second academic year or higher at an accredited Egyptian medical school. Students in their first academic year (due to the absence of a cumulative academic percentage), those who declined to provide informed consent, or those who submitted incomplete responses were excluded.

The required sample size was determined for a multivariable linear regression analysis. In our study, we treated the primary outcome (cumulative academic percentage) as a continuous variable to maximize statistical precision and avoid the loss of information inherent to categorization. To ensure the stability and reliability of the regression model, we followed the established rule of thumb recommending a minimum of 10 to 20 subjects per independent predictor (15). Given the 22 predictor terms included in our final model, this guideline indicated that a sample size of at least 220 to 440 participants was necessary. Therefore, our final sample of 751 participants comfortably exceeds this requirement, providing a robust foundation for the analysis.

### 2.3 Sampling and data collection approach

A convenience sampling approach was employed as the only feasible method for a national-level study of this scale, given the lack of a centralized, accessible registry of all medical students across Egypt, which is a prerequisite for probability-based sampling techniques like simple random sampling. To maximize reach and ensure representation from public, private, and Al-Azhar universities, the questionnaire was distributed electronically via social media and official online student forums. While this approach enabled broad recruitment, we acknowledge it introduces a potential for selection bias, as students who are more active online or engaged in student groups may be overrepresented. Data collection took place from January to May 2025. All responses were recorded anonymously to ensure participant privacy and data protection.

### 2.4 Study tool

Data were collected using a structured, self-administered online questionnaire adapted from a study by Jaber et al. (7) that explored socioeconomic disparities and academic attainment among medical students at Sudanese universities. The questionnaire used in that study was developed based on criteria from previously published research on the topic (9, 16). The final instrument was organized into three main sections.

The first section collected data on participants' demographics and institutions, including age, gender, academic year, university and university type, region, accommodation type, and urban or rural residence. The second section evaluated socioeconomic status and parental factors. This included items on perceived family income adequacy, the education levels and working status of both the father and mother, and the student's perception of parental support and whether their parents' education level affected their educational attitude. The final section assessed academic performance and history. This part of the questionnaire gathered self-reported data on the students' secondary school certificate score, their lowest degree obtained in previous exams, and whether they had ever failed a semester or been denied entry to an exam. The student's self-reported cumulative percentage from the last semester was used as the primary outcome measure of academic achievement.

### 2.5 Statistical analysis

Data was analyzed using Jamovi (Version 2.6). Descriptive statistics were used to summarize the socio-demographic and academic characteristics of the participants. Categorical variables were presented as frequencies and percentages, while continuous variables were described using means and standard deviations (SD).

The primary outcome was the “cumulative percentage last semester,” treated as a continuous variable. A two-stage linear regression modeling approach was employed to identify its predictors. First, a series of simple (univariate) linear regression analyses was performed for each potential predictor to assess its unadjusted association with academic performance. Subsequently, a multivariable linear regression model was constructed using a forced-entry method by entering all pre-selected, theoretically relevant independent variables into the model simultaneously to evaluate their independent effect while controlling for all other variables.

The selection of variables for the final model was based on established methodological principles to ensure validity and interpretability. Specifically, to avoid multicollinearity, the categorical “Family monthly income” variable was excluded in favor of the “Perceived Income” variable. Further, to prevent a tautological relationship (i.e., using a part of the outcome to predict the outcome itself), variables measuring academic performance or events were excluded because they are mechanically linked to the cumulative percentage. These included: “Percentage last semester,” “Cumulative Percentage last semester,” “Worst degree in a previous exam,” “Ever scored F (Fail),” and “Denied from entering an exam.”

The results of the regression models are presented as unstandardized beta coefficients (B) with their corresponding 95% confidence intervals (CI), as well as standardized beta (β) coefficients to allow for the comparison of effect sizes across predictors. A p-value of less than 0.05 was considered statistically significant.

## 3 Results

### 3.1 Participant characteristics

Detailed socio-demographic and academic characteristics are presented in Table 1. A total of 751 medical students in Egypt were included in the analysis. The mean age of participants was 20.9 years (SD = 1.40). The majority were female (55.9%), lived with their families (67.6%), and resided in urban areas (64.4%). Across academic years, the largest proportion of students was in their third year (33.8%). Most participants were enrolled in public universities (76.0%).

**Table 1:** Socio-demographic and academic characteristics of study participants (*N* = 751).

**Characteristic**		***N* (%)**
Age	Mean (SD)	20.9 (1.40)
Cumulative percentage	Mean (SD)	85.0 (8.96)
	Range (min–max)	25.0–100
Gender	Female	420 (55.9)
	Male	331 (44.1)
Academic year	2nd	166 (22.1)
	3rd	254 (33.8)
	4th	152 (20.2)
	5th	179 (23.8)
University type	Public	571 (76.0)
	Private/national	116 (15.4)
	Al-Azhar	64 (8.5)
Region	Urban Governorates	313 (41.7)
	Lower Egypt Governorates	286 (38.1)
	Upper Egypt	137 (18.2)
	Frontier Governorates	15 (2.0)
Accommodation	With Family	508 (67.6)
	In Student housing	243 (32.4)
Residence	Urban	484 (64.4)
	Rural	267 (35.6)
Perceived family income	Adequate	403 (53.7)
	Adequate with reserve	192 (25.6)
	Inadequate	156 (20.8)
Father's level of education	University	571 (76.0)
	Secondary school	141 (18.8)
	Primary school	39 (5.2)
Mother's level of education	University	523 (69.6)
	Secondary school	180 (24.0)
	Primary school	48 (6.4)
Parents' education affects attitude	Yes	394 (52.5)
	No	357 (47.5)
Parental support	Yes	676 (90.0)
	No	75 (10.0)
Father's working status	Clerk	464 (61.8)
	Manual working	224 (29.8)
	Not-working	63 (8.4)
Mother's working status	Not-working	398 (53.0)
	Clerk	275 (36.6)
	Manual working	78 (10.4)
Secondary school score	More than 90%	591 (78.7)
	Less than 90%	160 (21.3)
Worst degree in previous exams	< 65% (F)	93 (12.4)
	65–75% (C)	221 (29.4)
	76–85% (B)	234 (31.2)
	86–90% (A)	122 (16.2)
	>90% (A+)	81 (10.8)
Ever failed a semester	No	627 (83.5)
	Yes	124 (16.5)
Denied from entering exam	No	695 (92.5)
	Yes	56 (7.5)

SD, standard deviation.

Regarding parental background, most students reported having fathers (76.0%) and mothers (69.6%) with a university-level education, and most (90.0%) perceived their parents as supportive of their learning process. Regarding prior and ongoing academic performance, most participants (78.7%) entered medical school with a secondary school score above 90%, although 16.5% reported having failed a semester during their studies.

### 3.2 Factors associated with cumulative academic percentage

The overall cumulative percentage of all participants was 85.0 (SD = 8.96). Factors significantly associated with students' academic performance in univariable analysis are shown in Table 2, with multivariable analysis presented in Table 3. Multivariable linear regression analysis identified several factors significantly associated with students' academic performance. Male students had significantly lower cumulative percentages than female students (*B* = −1.81 [95% CI: −3.07, −0.54], β = −0.20 [95% CI: −0.34, −0.06]). Institutional and academic factors were also significant predictors. Students at Al-Azhar universities demonstrated lower academic performance than those at public universities (*B* = −5.84 [95% CI: −8.36, −3.32], β = −0.65 [95% CI: −0.94, −0.37]). Compared to second-year students, being in the fourth academic year was associated with lower scores (*B* = −3.04 [95% CI: −5.34, −0.74], β = −0.34 [95% CI: −0.60, −0.08]). Furthermore, living in student housing was associated with lower cumulative percentages compared to living with family (*B* = −3.09 [95% CI: −4.46, −1.72], β = −0.35 [95% CI: −0.50, −0.19]). Regarding geographical and social factors, students from Lower Egypt Governorates had higher cumulative percentages than those from Urban Governorates (*B* = 1.55 [95% CI: 0.07, 3.03], β = 0.17 [95% CI: 0.01, 0.34]). Finally, parental support during the learning process was associated with higher academic performance (*B* = 2.31 [95% CI: 0.29, 4.33], β = 0.26 [95% CI: 0.03, 0.48]).

**Table 2:** Univariate linear regression analysis of predictors for cumulative percentage among medical students in Egypt.

**Characteristic**	**Category**	**Cumulative percentage^1^**	**Univariate regression**		
			**Unstandardized Beta (95% CI)**	**Standardized Beta (95% CI)**	***p*** **-value**
Age	(continuous)		−0.33 (−0.79, 0.13)	−0.05 (−0.12, 0.02)	0.157
Gender	Female	86.5 (7.98)	Reference		
	Male	83.0 (9.72)	−3.54 (−4.81, −2.27)	−0.40 (−0.54, −0.25)	**< 0.001**
Academic year	2nd	86.9 (8.59)	Reference		
	3rd	84.1 (9.85)	−2.82 (−4.55, −1.08)	−0.31 (−0.51, −0.12)	**0.002**
	4th	83.1 (9.64)	−3.85 (−5.80, −1.89)	−0.43 (−0.65, −0.21)	**< 0.001**
	5th	86.1 (6.62)	−0.85 (−2.72, 1.03)	−0.10 (−0.30, 0.12)	0.375
University type	Public	85.8 (8.68)	Reference		
	Al-Azhar	76.6 (9.18)	−9.13 (−11.36, −6.90)	−1.02 (−1.27, −0.77)	**< 0.001**
	Private/national	85.8 (7.83)	0.08 (−1.64, 1.79)	0.01 (−0.18, 0.20)	0.932
Region	Urban Governorates	83.3 (9.25)	Reference		
	Frontier Governorates	82.0 (9.46)	−1.32 (−5.90, 3.25)	−0.15 (−0.66, 0.36)	0.571
	Lower Egypt Governorates	86.9 (8.82)	3.57 (2.16, 4.99)	0.40 (0.24, 0.56)	**< 0.001**
	Upper Egypt	85.2 (7.68)	1.87 (0.10, 3.65)	0.21 (0.01, 0.41)	**0.038**
Accommodation	With family	86.6 (8.43)	Reference		
	Student housing	81.7 (9.12)	−4.91 (−6.24, −3.59)	−0.55 (−0.70, −0.40)	**< 0.001**
Residence	Urban	85.4 (8.80)	Reference		
	Rural	84.2 (9.20)	−1.15 (−2.49, 0.19)	−0.13 (−0.28, 0.02)	0.093
Perceived income	Adequate	85.0 (8.65)	Reference		
	Adequate with reserve	86.6 (8.41)	1.59 (0.07, 3.11)	0.18 (0.01, 0.35)	**0.038**
	Inadequate	82.9 (9.95)	−2.33 (−3.99, −0.66)	−0.26 (−0.45, −0.07)	**0.011**
Secondary school score	More than 90%	85.2 (8.65)	Reference		
	Less than 90%	84.2 (9.99)	−1.05 (−2.62, 0.52)	−0.12 (−0.29, 0.06)	0.188
Father's education	University	85.3 (8.53)	Reference		
	Primary school	85.3 (9.06)	0.01 (−2.90, 2.91)	0.00 (−0.32, 0.33)	0.997
	Secondary school	83.7 (10.44)	−1.62 (−3.27, 0.04)	−0.18 (−0.37, 0.00)	0.055
Mother's education	University	85.8 (8.31)	Reference		
	Primary school	82.6 (9.60)	−3.21 (−5.84, −0.58)	−0.36 (−0.65, −0.07)	**0.017**
	Secondary school	83.1 (10.17)	−2.69 (−4.19, −1.18)	−0.30 (−0.47, −0.13)	**< 0.001**
Parental education affects attitude	No	84.7 (9.18)	Reference		
	Yes	85.3 (8.75)	0.61 (−0.68, 1.89)	0.07 (−0.08, 0.21)	0.353
Parental support	No	83.1 (11.2)	Reference		
	Yes	85.2 (8.65)	2.14 (0.00, 4.27)	0.24 (0.00, 0.48)	**0.05**
Father's work status	Clerk	85.3 (8.84)	Reference		
	Manual working	84.3 (9.02)	−1.03 (−2.46, 0.40)	−0.12 (−0.28, 0.04)	0.157
	Not-working	84.8 (9.55)	−0.54 (−2.91, 1.82)	−0.06 (−0.32, 0.20)	0.651
Mother's work status	Not-working	84.2 (9.22)	Reference		
	Clerk	85.9 (8.45)	1.65 (0.28, 3.02)	0.18 (0.03, 0.34)	**0.019**
	Manual working	85.8 (9.08)	1.61 (−0.56, 3.78)	0.18 (−0.06, 0.42)	0.147

Bold values indicate statistical significance (p < 0.05). 1 Values are presented as Mean (Standard Deviation).

**Table 3:** Multivariable linear regression analysis of predictors for cumulative percentage among medical students in Egypt.

**Characteristic**	**Category**	**Cumulative percentage^1^**	**Multivariable regression**		
			**Unstandardized beta (95% CI)**	**Standardized beta (95% CI)**	***p*** **-value**
Age	(continuous)		−0.31 (−1.06, 0.44)	−0.05 (−0.17, 0.07)	0.417
Gender	Female	86.5 (7.98)	Reference		
	Male	83.0 (9.72)	−1.81 (−3.07, −0.54)	−0.20 (−0.34, −0.06)	**0.005**
Academic year	2nd	86.9 (8.59)	Reference		
	3rd	84.1 (9.85)	−1.51 (−3.31, 0.29)	−0.17 (−0.37, 0.03)	0.1
	4th	83.1 (9.64)	−3.04 (−5.34, −0.74)	−0.34 (−0.60, −0.08)	**0.01**
	5th	86.1 (6.62)	−0.41 (−3.46, 2.65)	−0.05 (−0.39, 0.30)	0.793
University type	Public	85.8 (8.68)	Reference		
	Al-Azhar	76.6 (9.18)	−5.84 (−8.36, −3.32)	−0.65 (−0.94, −0.37)	**< 0.001**
	Private/national	85.8 (7.83)	1.74 (−1.05, 4.54)	0.20 (−0.12, 0.51)	0.221
Region	Urban Governorates	83.3 (9.25)	Reference		
	Frontier Governorates	82.0 (9.46)	−1.96 (−6.35, 2.43)	−0.22 (−0.71, 0.27)	0.381
	Lower Egypt Governorates	86.9 (8.82)	1.55 (0.07, 3.03)	0.17 (0.01, 0.34)	**0.04**
	Upper Egypt	85.2 (7.68)	−1.04 (−3.70, 1.62)	−0.12 (−0.41, 0.18)	0.443
Accommodation	With family	86.6 (8.43)	Reference		
	Student housing	81.7 (9.12)	−3.09 (−4.46, −1.72)	−0.35 (−0.50, −0.19)	**< 0.001**
Residence	Urban	85.4 (8.80)	Reference		
	Rural	84.2 (9.20)	−0.30 (−1.64, 1.05)	−0.03 (−0.18, 0.12)	0.664
Perceived income	Adequate	85.0 (8.65)	Reference		
	Adequate with reserve	86.6 (8.41)	1.15 (−0.30, 2.59)	0.13 (−0.03, 0.29)	0.119
	Inadequate	82.9 (9.95)	−1.23 (−2.83, 0.38)	−0.14 (−0.32, 0.04)	0.133
Secondary school score	More than 90%	85.2 (8.65)	Reference		
	Less than 90%	84.2 (9.99)	−1.52 (−3.19, 0.15)	−0.17 (−0.36, 0.02)	0.074
Father's education	University	85.3 (8.53)	Reference		
	Primary school	85.3 (9.06)	2.30 (−1.55, 6.15)	0.26 (−0.17, 0.69)	0.24
	Secondary school	83.7 (10.44)	0.15 (−1.80, 2.10)	0.02 (−0.20, 0.23)	0.881
Mother's Education	University	85.8 (8.31)	Reference		
	Primary school	82.6 (9.60)	−0.77 (−4.30, 2.76)	−0.09 (−0.48, 0.31)	0.668
	Secondary school	83.1 (10.17)	−0.98 (−2.80, 0.85)	−0.11 (−0.31, 0.10)	0.296
Parental education affects attitude	No	84.7 (9.18)	Reference		
	Yes	85.3 (8.75)	−0.16 (−1.47, 1.14)	−0.02 (−0.16, 0.13)	0.806
Parental support	No	83.1 (11.2)	Reference		
	Yes	85.2 (8.65)	2.31 (0.29, 4.33)	0.26 (0.03, 0.48)	**0.025**
Father's work status	Clerk	85.3 (8.84)	Reference		
	Manual working	84.3 (9.02)	−1.02 (−2.61, 0.56)	−0.11 (−0.29, 0.06)	0.204
	Not-working	84.8 (9.55)	0.17 (−2.09, 2.43)	0.02 (−0.23, 0.27)	0.881
Mother's work status	Not-working	84.2 (9.22)	Reference		
	Clerk	85.9 (8.45)	0.24 (−1.24, 1.72)	0.03 (−0.14, 0.19)	0.75
	Manual working	85.8 (9.08)	1.36 (−0.84, 3.56)	0.15 (−0.09, 0.40)	0.224

Bold values indicate statistical significance (p < 0.05). 1 Values are presented as Mean (Standard Deviation).

## 4 Discussion

The relationship between socioeconomic status and academic performance is a critical topic in educational research. However, the specific determinants of success vary significantly across different cultural and educational systems, and studies exploring these predictors in Egypt are scarce. This study aimed to fill this gap by identifying factors influencing academic achievement among Egyptian medical students. While our convenience sampling method means the findings may not be fully generalizable to all medical students in Egypt, the results from our large, multi-institutional cohort provide valuable initial insights. Furthermore, we acknowledge that the use of self-reported cumulative percentages is a significant limitation. This approach is susceptible to both recall bias and social desirability bias, which may have resulted in an overestimation of the mean academic performance (85.0%). This potential measurement error could attenuate the true strength of the observed associations, as random inaccuracies in self-reporting can make it more difficult to detect genuine effects. Nevertheless, our findings highlight that, within this sample, institutional and demographic characteristics appear to be independent predictors of academic performance, alongside traditional socioeconomic indicators.

A total of 751 respondents were included in our study, with a notable female preponderance (55.9%). Their mean cumulative percentage was 85%. Our participants were recruited from 38 institutions from different regions in Egypt, with students from urban governorates representing 41.7% of the sample. Regarding the sociodemographic features of the parents, 69.6% of mothers and 76% of fathers had a university degree. An adequate family income was reported by 53.7% of participants, 90% of whom received parental financial assistance.

Our linear regression analysis revealed significant predictors of medical students' cumulative percentage. Al-Azhar university students had significantly lower academic performance compared to students in public universities. A plausible hypothesis for this disparity is the unique dual-curriculum system at Al-Azhar. We speculate that the requirement to study religious subjects alongside an extensive medical curriculum could impose a substantially heavier academic load, potentially impacting performance in medical courses. Regardless of the cause, however, this performance gap has substantial implications for the equity of postgraduate residency selection in Egypt. The current system directly compares student scores from different institutions without accounting for systemic variability in curricula and assessment rigor. This could significantly disadvantage otherwise capable students from institutions with more demanding academic programs, ultimately affecting their career trajectories.

Beyond institutional factors, our study observed the critical role of a student's immediate social environment and family support system. Although participants from upper Egypt had better cumulative percentages in the univariate analysis (*p* = 0.038), the significance of this finding was lost in the multivariate analysis (*p* = 0.443). Conversely, students from governorates in lower Egypt had exceedingly better scores (*p* = 0.009) compared to those in urban governorates. Other factors that were associated with poor academic performance included living in student housing (*p* < 0.001) and inadequate family income (*p* = 0.011). On the other hand, receiving parental support was associated with significantly better academic performance (*p* = 0.025).

In a systematic review by Kassaw and Demareva, the lower academic performance of students who live in dormitories was related to the crowdedness which is not a suitable environment for studying. In addition, they highlighted inadequate social support as a key factor in poor educational outcomes (6). Nevertheless, our findings conflict with those of a study on Ethiopian medical students, which found that being nondormitory was associated with poor performance (8). Furthermore, a study of Sudanese medical students found that living conditions had no significant impact on cumulative GPA (7). Thus, perhaps a more compelling interpretation for our findings is that students in university housing may struggle with the demanding workload of medical education without the immediate psychological and logistical support their families provide. In a qualitative study among medical students in Iran, family and social support were reported to be key facilitators of academic achievement among medical students (17). On the other hand, parental support was not significantly associated with cumulative GPA in two studies among medical students in Sudan (7, 9), suggesting the impact of parental support can vary across different cultural settings.

Our study presents a nuanced perspective on parental influence, distinguishing between functional support and structural socioeconomic status. While perceived family income demonstrated a significant association with academic scores in the univariate analysis, this effect became non-significant in the final multivariable model after adjusting for other factors. In contrast, direct parental support remained a significant predictor of higher academic achievement. This suggests that for Egyptian medical students who have already navigated the rigorous high school selection process for medical school, the functional, day-to-day support they receive from their families may be more critical to their ongoing success than the structural advantages conferred by financial status alone.

This interpretation is further supported by a recent nationwide study of Egyptian medical students (12). While their research focused on academic self-efficacy rather than direct academic grades, it found that financial stability was a significant predictor of higher academic self-efficacy. These findings suggest that while financial security may bolster a student's confidence, the tangible, functional support from family translates more directly into higher academic grades in this cohort. However, this contrasts with a study on Sudanese medical students, which found that high family income remained a significant predictor of higher cumulative GPA (7). This variability suggests that the impact of socioeconomic status is not uniform and may be reshaped by the students' social environments. In the work of Guerrero-Lopez et al., the academic performance of medical students had weak, but significant, positive associations with their financial conditions (*r* = 0.241), interpersonal relationships with roommates (*r* = 0.165), and interpersonal relationships with other peers (*r* = 0.217) (18).

In our multivariable analysis, male students had significantly lower cumulative percentages than their female counterparts (*p* = 0.005). This contrasts with several other regional studies on academic performance, including those in Sudan and in Ethiopia (7, 8), which found no significant difference between genders. Our finding, however, becomes more intriguing when interpreted in light of recent large-scale studies in Egypt, which found that male Egyptian medical students reported significantly lower stress and higher psychological resilience than females (19). Similarly, a nationwide study by Samir et al., though without statistical significance, reported that male students had slightly higher academic self-efficacy scores (12). While Egyptian male students may report greater psychological resilience and academic confidence, these factors do not appear to translate into superior academic grades. This potential discrepancy suggests that other unmeasured variables, such as differences in study habits, engagement with the curriculum, or societal expectations of females, may be more powerful determinants of final academic performance than these psychological attributes alone.

Finally, a key and perhaps counterintuitive finding of this study is the absence of a significant association between traditional socioeconomic markers and academic performance in the final model. While many studies highlight parental education and family income as major determinants of student achievement (12, 16, 18), these factors did not emerge as significant predictors in this context once other variables were considered. This pattern suggests a distinct educational environment among Egyptian medical students, where immediate contextual and institutional factors may outweigh family background. The influence of university type, living arrangements, and parental involvement appears to shape academic outcomes more strongly than socioeconomic history. In the demanding and highly standardized setting of medical education in Egypt, factors such as institutional culture, curriculum intensity, and available support systems may therefore serve as more proximal determinants of student performance. These findings highlight the need to consider contextual influences when interpreting academic achievement rather than relying solely on conventional socioeconomic explanations.

### 4.1 Limitations

Nonetheless, this study has several limitations to consider when interpreting the results. For instance, the cross-sectional design can only establish associations between predictors and academic performance; it cannot determine causality. Furthermore, a convenience sampling approach was employed, which may introduce selection bias and limit the formal generalizability of our findings to all medical students in Egypt. Finally, the primary outcome was based on self-reported cumulative percentages, which is susceptible to both recall and social desirability biases, which may have led to an overestimation of the mean academic performance in our sample. Moreover, such measurement error can attenuate the true strength of the observed associations, potentially underestimating the effects of significant predictors.

## 5 Conclusions

Based on this large, multi-institutional cohort, our study suggests that academic performance among Egyptian medical students is determined by a complex interplay of institutional, demographic, and social support factors, which appear to be more influential than traditional socioeconomic markers. Specifically, male gender, Al-Azhar University enrollment, and student housing residence were independent predictors of lower academic performance, while direct parental support significantly predicted higher achievement. Although limited by the sampling method, these findings highlight potential systemic inequities, particularly as students from varied institutional backgrounds are ultimately compared against a single metric for postgraduate residency placement. Therefore, these results suggest a need for further investigation and a potential review of educational policies. Future studies using probability sampling would be valuable to confirm these findings and ensure a more equitable and meritocratic pathway for all medical graduates.

## Data Availability

The dataset analyzed in this study are available from the corresponding author on reasonable request.

## SES collaborative group

Mohamed Sayed Ramadan, Hala Gamal Waly, Noor Elshinawy, Fatma Gamal Ragab, Hadeer Elsayed Mira, Abdallah Nasr Alsaaidy and Shahd Sameh Ahmed Ragab.
